# Viral RNA is a target for *Wolbachia*-mediated pathogen blocking

**DOI:** 10.1371/journal.ppat.1008513

**Published:** 2020-06-18

**Authors:** Tamanash Bhattacharya, Irene L. G. Newton, Richard W. Hardy

**Affiliations:** Department of Biology, Indiana University, Bloomington, Indiana, United States of America; The University of Queensland, AUSTRALIA

## Abstract

The ability of the endosymbiont *Wolbachia pipientis* to restrict RNA viruses is presently being leveraged to curb global transmission of arbovirus-induced diseases. Past studies have shown that virus replication is limited early in arthropod cells colonized by the bacterium, although it is unclear if this phenomenon is replicated in mosquito cells that first encounter viruses obtained through a vertebrate blood meal. Furthermore, these cellular events neither explain how *Wolbachia* limits dissemination of viruses between mosquito tissues, nor how it prevents transmission of infectious viruses from mosquitoes to vertebrate host. In this study, we try to address these issues using an array of mosquito cell culture models, with an additional goal being to identify a common viral target for pathogen blocking. Our results establish the viral RNA as a cellular target for *Wolbachia-*mediated inhibition, with the incoming viral RNA experiencing rapid turnover following internalization in cells. This early block in replication in mosquito cells initially infected by the virus thus consequently reduces the production of progeny viruses from these same cells. However, this is not the only contributor to pathogen blocking. We show that the presence of *Wolbachia* reduces the per-particle infectivity of progeny viruses on naïve mosquito and vertebrate cells, consequently limiting virus dissemination and transmission, respectively. Importantly, we demonstrate that this aspect of pathogen blocking is independent of any particular *Wolbachia-*host association and affects viruses belonging to *Togaviridae* and *Flaviviridae* families of RNA viruses. Finally, consistent with the idea of the viral RNA as a target, we find that the encapsidated virion RNA is less infectious for viruses produced from *Wolbachia-*colonized cells. Collectively, our findings present a common mechanism of pathogen blocking in mosquitoes that establish a link between virus inhibition in the cell to virus dissemination and transmission.

## Author summary

Viruses transmitted by arthropod vectors pose a significant global health risk. Incidence of diseases caused by these viruses can thus be reduced by implementing effective vector control strategies. This need is further exacerbated due to the lack of commercially available vaccines and antivirals. Presence of the intracellular bacteria *Wolbachia pipientis* is associated with virus inhibition in multiple mosquito vectors. Furthermore, *Wolbachia* is inherited transovarially and spreads across the vector population like a natural gene drive, making it an attractive vector control agent. In this study, we examine how the presence of the bacterium in arthropod cells prevents initial establishment of vertebrate cell derived viruses. Our results indicate rapid turnover of incoming viral RNA very early during infection in *Wolbachia-*colonized cells, thus establishing it as a cellular target for pathogen blocking. Additionally, upon evaluating how these events might further limit virus spread, we find that infectivity of progeny viruses belonging to multiple RNA virus families are reduced on a per-particle basis. This aspect of virus inhibition is independent of any particular *Wolbachia-*host association and affects how these viruses replicate in naïve mosquito and vertebrate cells, thus providing a collective basis of reduced virus dissemination and transmission in *Wolbachia-*colonized mosquitoes.

## Introduction

The pathogen blocking ability of the arthropod endosymbiont *Wolbachia pipientis* makes it an exciting biocontrol agent that is currently being used to limit transmission of arboviruses around the world [1]. The importance of studying the underlying mechanism of pathogen blocking, however, is not only to determine the long-term feasibility of this strategy, but to also learn how this particular arthropod host-endosymbiont association enables the former to become refractory to a wide range of RNA viruses. The degree of virus inhibition varies between different *Wolbachia-*host associations and is dependent on the *Wolbachia* strain and the arthropod host species [2–5]. Collectively, three major aspects of pathogen blocking have been reported consistently across all associations: inhibition of viruses possessing positive-sense single-stranded RNA (+ ssRNA) genomes, limited virus dissemination, and lower virus transmission [2, 6–9]. Earlier studies have primarily concentrated on identifying cellular events that lead to virus inhibition in arthropod cells, including viral entry, genome replication and protein translation [3, 10–14]. Indeed, this approach has proven to be very useful in identifying important host determinants of pathogen blocking. However, too often results from these studies are limited to a single *Wolbachia-*host association, giving the impression that different *Wolbachia-*host permutations involve distinct mechanisms of inhibition [3, 10–13]. Furthermore, these studies do not address the question of how virus inhibition in *Wolbachia-*colonized cells ultimately limit virus dissemination within mosquito tissues, and transmission from mosquitoes to vertebrates. To this end, the goal of this study was to utilize an array of mosquito cell culture models representing different *Wolbachia-*host combinations to identify a common viral target for pathogen blocking and to determine the link between intracellular virus inhibition, restricted virus dissemination and virus transmission. Our results identify the viral RNA genome as a target for pathogen blocking in arthropods, which include both fruit flies and mosquitoes. In *Wolbachia-*colonized cells, viral RNA targeting occurs at multiple stages of the replication cycle, notably in the very early stages following virus internalization and genome delivery, that lead to a shortened half-life of the incoming viral RNA. Additionally, we demonstrate that viral RNA present within viruses produced from *Wolbachia-*colonized cells are less infectious in vertebrate cells, contributing to the overall reduced infectivity of the progeny viruses. We provide evidence that this aspect of pathogen blocking is likely independent of any particular *Wolbachia* strain and arthropod host association and affects members of at least two + ssRNA virus families. Finally, we show that two major aspects of pathogen blocking i.e. limited virus dissemination and transmission occur due to the inability of these less infectious viruses to propagate in naïve arthropod and vertebrate cells.

## Results

### Viral RNA is a shared target for multi-stage inhibition in *Wolbachia-*colonized cells

**Fig 1:**
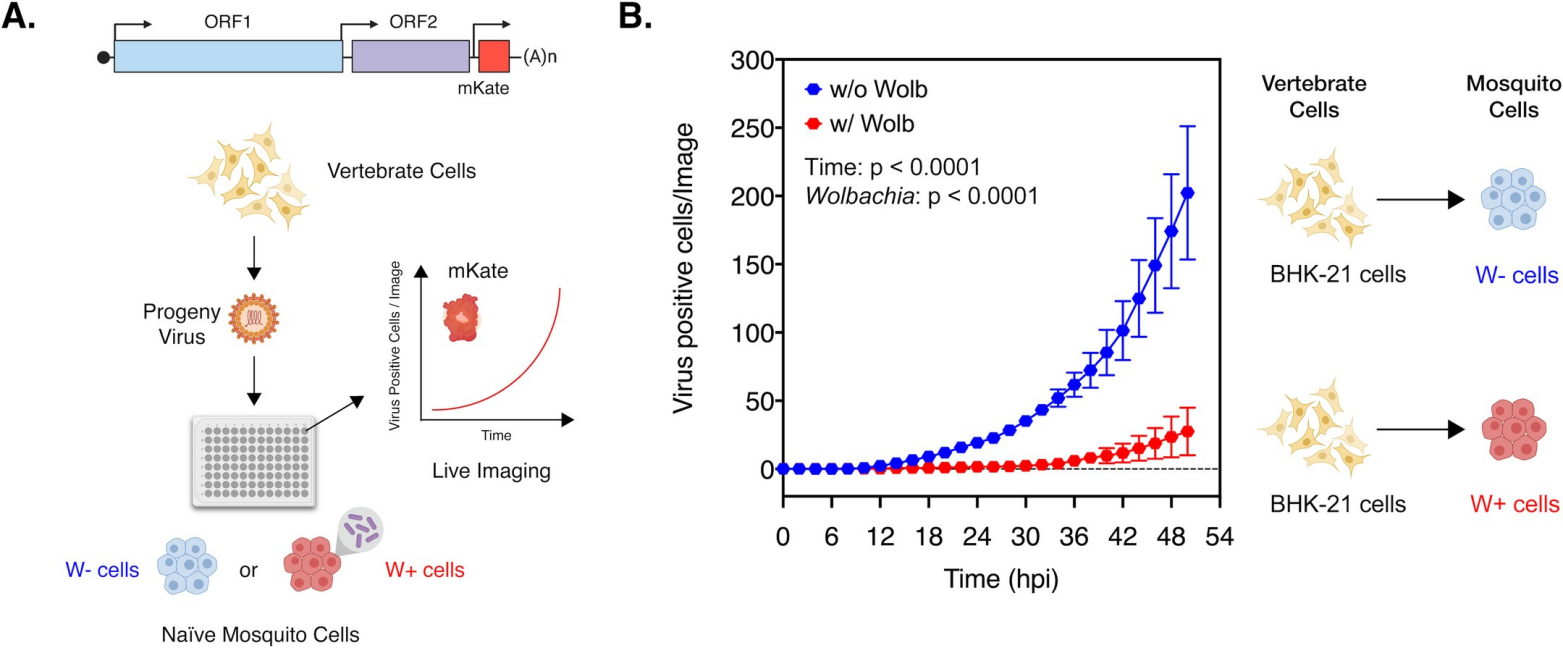
Presence of *Wolbachia* reduces spread of vertebrate-derived viruses in naïve mosquito cells. (A) Schematic representation of the experiment. CHIKV expressing mKate fluorescent protein from a second sub-genomic promoter was grown in BHK-21 cells. These progeny viruses were then used to infect naïve C710 cells with (depicted in red) and without (depicted in blue) *Wolbachia* (*w*Stri strain) synchronously at an MOI of 1 particle/cell. Virus growth in cells, plated on a ninety-six-well plate, was measured in real time by imaging and quantifying the number of red cells (Virus Positive Cells/Image) expressing the virus encoded mKate protein over a period of 48 hours, using live cell imaging. (B) Color of the data points distinguish the two destination cell lines where virus replication was assayed on; blue represent C710 cells without *Wolbachia* while red represent C710 cells with *Wolbachia*. Y-axis label (Virus Positive Cells/Image) represent red cells expressing virus-encoded mKate fluorescent protein in a single field of view, four of which were averaged/sample at every two-hour time point collected over the course of infection. Two-way ANOVA with Sidak’s post-hoc test. Error bars represent standard error of mean (SEM) of biological replicates (n = 7–9).

Presence of *Wolbachia* is associated with reduced viral gene expression in arthropod cells. This widely reported aspect of virus inhibition can be observed both *in vivo*, in *Aedes aegypti* mosquitoes colonized with *w*AlbB (S1A Fig; Welch-corrected unpaired two-tailed t-test, p = 0.0256, t = 2.989, df = 5.749) as well as in cell culture, *Aedes albopictus* cells colonized with *w*Mel (S1B Fig; Welch-corrected unpaired two-tailed t-test, p < 0.0001, t = 55.23, df = 5.712) [10–12, 14]. However, this quantification represents total viral RNA accumulated over multiple rounds of virus replication in mosquito cells. To determine how inhibition occurs in the initial stages of infection in the vector i.e. during establishment of viruses acquired through a blood meal, we monitored the spread of vertebrate cell-derived viruses in naïve *Aedes albopictus* mosquito cells (C710 cells) colonized with and without *Wolbachia* derived from the planthopper host, *Laodelphax striatellus* (*w*Stri). It should be noted that throughout this study, multiplicity of infection (MOI) was calculated in terms of viral genome equivalents (particles) instead of infectious units (PFUs), given that the former is independent and the latter is dependent on a single host/tissue cell line (e.g. vertebrate or arthropod) that the viruses are assayed on to determine virus “infectivity”. Spread of Chikungunya viruses derived from BHK-21 (Baby Hamster Kidney fibroblast cells) expressing a fluorescent reporter protein (CHIKV-mKate) was thus assessed in these mosquito cells following a synchronized infection at a low MOI (1 particle/cell) that involved virus adsorption at 4ºC. Cell monolayers were then extensively washed with 1XPBS to remove any unbound viruses and warm media (37ºC) was added to cells to initialize virus internalization and infection. Virus spread was then measured over 50 hours by quantifying mean virus-encoded fluorescent reporter expression observed over four distinct fields of view taken per well every 2 hours (Fig 1A). Two-way ANOVA was used to determine the effect of destination cell type (with or without *w*Stri) and/or time on virus spread. Virus growth was significantly reduced over time in cells colonized with *w*Stri compared to cells without the bacterium; Ordinary Two-way ANOVA with Sidak’s multiple comparisons test, *Wolbachia*: p < 0.0001, Time: p < 0.0001, Time X *Wolbachia*: p < 0.0001 (Fig 1).

Although our previous result validates the initial stage of virus inhibition in the vector, i.e. during virus establishment in *Wolbachia-*colonized mosquito tissues following an infectious blood meal, it is unknown how early this inhibition takes place at the cellular level in cells initially infected with the virus. Previous studies by other groups have indicated that inhibition of both alphaviruses and flaviviruses occur at an early stage of infection in *Wolbachia-*colonized arthropod cells [12–13]. Furthermore, data from the latter study indicate that viral RNA is susceptible to degradation immediately post-internalization [12]. Therefore, to determine whether viral RNA is degraded faster in *Wolbachia-*colonized cells following virion internalization, we asked if the incoming viral RNA half-life is altered between cells with and without *Wolbachia*. We also asked whether or not this event is exclusive to mosquito cells.

**Fig 2:**
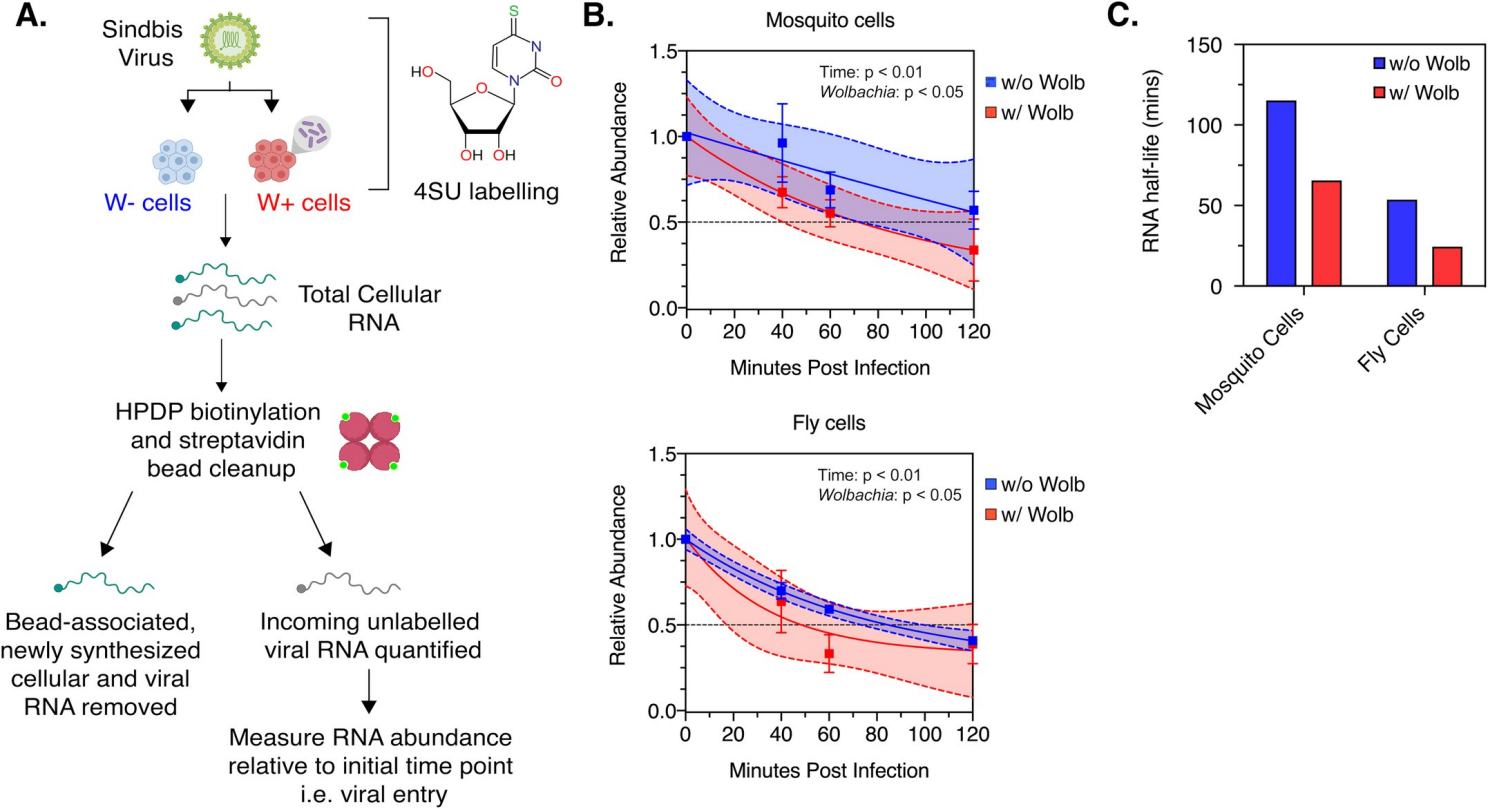
Incoming viral RNA is degraded quicker in *Wolbachia-*colonized cells. Half-life of incoming viral RNA was assessed following infection of C710 *Aedes albopictus* cells colonized with and without *Wolbachia* (*w*Stri strain) and JW18 *Drosophila melanogaster* cells with and without *Wolbachia* (*w*Mel strain). (A) Schematic representation of the experiment. Sindbis virus derived from vertebrate BHK-21 cells was used to synchronously infect C710 or JW18 cells pre-labelled with 50 μM 4-thiouridine (4SU) for 12 hours. Infection was carried out under labelling conditions. At indicated times post infection, total RNA was extracted from cells, biotinylated and streptavidin beads were used to isolate incoming unlabeled viral RNA from 4SU-labelled cellular and newly synthesized RNA. Viral RNA was quantified at each time-point using quantitative RT-PCR relative to viral RNA detected at the start of infection (0h). (B) Relative abundance of incoming viral RNA in cells colonized with and without *Wolbachia* over 120 minutes post-infection were determined by qRT-PCR analysis as described in Materials and Methods. Non-linear, exponential regression analyses was performed to determine the viral RNA decay profile (represented by solid lines). Dashed lines represent the 95% CI of the aforementioned regression. Two-way ANOVA with Sidak’s post-hoc test. Error bars represent standard error of mean (SEM) of biological replicates (n = 3) (C) Viral RNA half-lives were estimated from data showed in (B).

C710 *Aedes albopictus* cells with (*w*Stri strain) or without *Wolbachia* (w/o *w*Stri) and JW18 *Drosophila melanogaster* cells with (*w*Mel strain) or without *Wolbachia* (w/o *w*Mel) were grown overnight in media containing the cross-linkable nucleoside analog 4-thiouridine (4SU) at 50 μM (Fig 2A). In the cell, 4SU is converted to 4S-UTP before being incorporated into newly synthesized RNA, thus allowing labelling of all cellular RNA. Sindbis virus (SINV) derived from vertebrate BHK-21 cells grown in normal media, and therefore containing an unlabeled virion RNA genome, was then used to synchronously infect the 4SU-treated cells at an high MOI of 10 particles/cell. Following virus adsorption at 4ºC, cell monolayers were extensively washed with 1XPBS to remove any unbound viruses. Warm media (37ºC) containing 4SU was added to cells to initialize virus internalization and infection was then carried out under labelling conditions, thus allowing 4S-UTP incorporation into newly synthesized host and viral RNA, leaving the incoming viral RNA as the only unlabeled RNA species in the cell. All 4SU-labelled RNA was separated from the total RNA pool following biotinylation and streptavidin cleanup. Next, levels of unlabeled RNA at each time point was measured relative to that present at 0 minutes post internalization, which represent initial viral genome delivery. RNA half-life was extrapolated using non-linear, exponential decay model (Fig 2B). We observed steady monophasic decay of the incoming viral RNA in both mosquito and fly cells, either in the presence or absence of *Wolbachia*. Two-way ANOVA with Sidak’s post-hoc test revealed an effect of both time and *Wolbachia* on the relative abundance of incoming viral RNA; C710 mosquito cells, Time: p = 0.004, *Wolbachia* (*w*Stri) = 0.0469, JW18 fly cells, Time: p = 0.0042, *Wolbachia* (*w*Mel) = 0.021. (Fig 2B). However, in each case mean half-life of viral RNA was reduced in *Wolbachia-*colonized cells. In mosquito cells, RNA half-life was reduced approximately 1.8-fold; One-phase decay, 115.2 minutes (w/o *w*Stri), 65.58 minutes (w/ *w*Stri). In comparison, half-life was reduced approximately 2.2-fold in fly cells; 53.7 minutes (w/o *w*Mel), 24.4 minutes (w/ *w*Mel) (Fig 2C). Based on these results, we conclude that the incoming viral RNA undergoes faster turnover in *Wolbachia-*colonized cells, reducing the cellular pool of viral RNA very early in the replication cycle. Abrogation of both viral RNA synthesis and protein expression have been previously demonstrated in the presence of *Wolbachia* [10–14]. However, our current data alongside observations made by Thomas et.al, further support the idea of viral RNA as a cellular target for pathogen blocking at multiple steps of the viral infection cycle [13].

### Progeny viruses generated from *Wolbachia* colonized cells are less infectious

It is unclear how the aforementioned cellular events lead to reduced virus dissemination within the mosquito vector as well as reduced transmission into vertebrate hosts. In our previous study, we reported reduced infectivity of Sindbis viruses derived from *Wolbachia-*colonized fly cells on vertebrate BHK-21 cells [12]. In light of this result, we wondered whether these progeny viruses are compromised in their ability to infect and propagate in naïve vertebrate and arthropod cells. We reasoned that lower production of total viruses from *w*Stri*-*colonized C710 mosquito cells in combination with their inability to propagate in naïve C710 mosquito cells might explain why virus dissemination is reduced in mosquitoes carrying *Wolbachia*. Additionally, the observed loss in transmission could occur as a result of these viruses being unable to spread and kill vertebrate cells. But given that *Wolbachia*-mediated reduction of virus dissemination and transmission has been observed against multiple RNA viruses across multiple *Wolbachia*-host associations, we first wanted to expand our previous findings to determine whether our previous observation regarding the loss in per-particle infectivity is limited either to any particular virus or certain *Wolbachia-*host associations [2, 6–9, 12].

**Fig 3:**
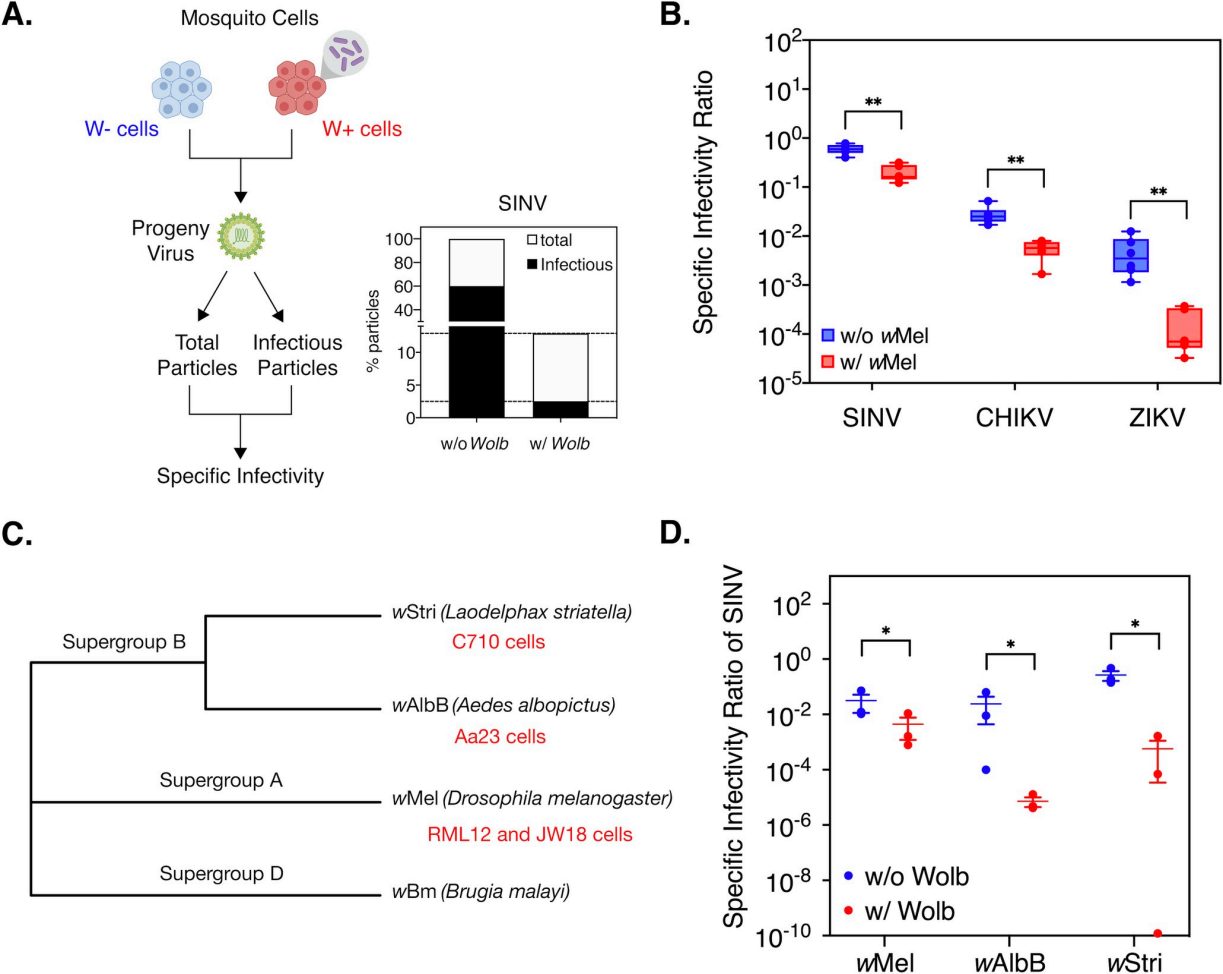
Presence of *Wolbachia* in mosquito cells reduces progeny virus infectivity in vertebrate cells. RML12 mosquito cells with and without *Wolbachia* (*w*Mel strain) were infected with alphaviruses Sindbis (SINV), Chikungunya (CHIKV) or flavivirus Zika (ZIKV) at an MOI of 10 particles/cell. Viral supernatants were harvested at 48 hours post infection. Infectious virus titer was quantified by performing plaque assays on BHK-21 (SINV, CHIKV) and Vero (ZIKV) cells. Total virus particles were determined by quantifying viral genome copies present in the supernatant using qRT-PCR. Reported specific infectivity (SI) ratios were calculated as total infectious virus titer divided by total particles produced per mL of viral supernatant. (A) Percentage of total SINV particles produced from cells with and without *Wolbachia* that are infectious on BHK-21 cells. (B) Specific Infectivity Ratios of progeny SINV, CHIKV and ZIKV viruses (n = 6). Welch’s t-test performed on log-transformed values. (C) Maximum-likelihood tree representing the phylogenetic relationship between the *Wolbachia* strains used in the study was generated using MEGA X, using a MUSCLE alignment of concatenated sequences of multi-locus typing (MLST) genes (*coxA*, *gatB*, *ftsZ*, *hcpA*, *fbpA)*. Sequences from *Wolbachia* strain *w*Bm, native to the filarial nematode *Brugia malayi*, was used as a distant outgroup. Corresponding host cell lines used in this study are indicated in red below their respective *Wolbachia* strain tip labels. (D) Specific Infectivity of progeny SINV derived from *Aedes albopictus* cells colonized with non-native (*w*Mel and *w*Stri) and native (*w*AlbB) *Wolbachia* strains. Cells were infected with virus at an MOI of 0.1 particles/cell and infectious virus titer produced after 48 hours was quantified via plaque assays on BHK-21 cells. Error bars represent standard error of mean (SEM) of biological replicates (n = 3). One-way ANOVA with Tukey’s post-hoc test performed on log-transformed values. *P < 0.05, **P < 0.01.

Viruses relevant to our study i.e. alphaviruses and flaviviruses are not known to form empty virions [15]. Thus, all virus particles produced from cells, regardless of infectivity, contain viral RNA cargo that can be measured using quantitative PCR. We therefore quantified viral genome copies present in the cell supernatant as a proxy for total virus particles released following infection in an arthropod host i.e. *Aedes albopictus-*derived or *Drosophila melanogaster-*derived cells with and without *Wolbachia*. Infectious viruses present in the same cell supernatant were assayed by quantifying plaque-forming units on vertebrate cells. Following independent quantification these attributes in tandem, we then calculated per-particle infectivity or specific infectivity ratio (SI) of progeny viruses as the ratio of infectious virus to total virus (Fig 3A) [12, 16].

First, we assessed the ability of a single *Wolbachia* strain-type, *w*Mel, to inhibit multiple RNA viruses across two different arthropod host cell types, mosquito (*Aedes albopictus* RML12 cells) and the native fruit fly (*Drosophila melanogaster* JW18 cells). Mosquito cells were challenged with a panel of arboviruses, including alphaviruses Sindbis (SINV) and Chikungunya (CHIKV) and one flavivirus, Zika (ZIKV) to determine the breadth of this phenotype against multiple arboviruses. In all cases, viruses grown in the presence of *Wolbachia* (W+ viruses) exhibited a lower SI ratio than viruses grown in cells without the endosymbiont (W- viruses). Degree to which infectivity (SI) was reduced varied depending on the virus type; Welch-corrected unpaired two-tailed t-test on log-transformed values, SINV: 3-fold reduction (p = 0.000059, df = 10, t-ratio = 6.627), CHIKV: 5-fold reduction (t-test, p = 0.000071, df = 10, t-ratio = 6.6.471), ZIKV: 32-fold reduction (t-test, p = 0.000144, df = 10, t-ratio = 5.934) (Fig 3A and 3B, S2 Fig). We obtained similar results when we tested the virus panel in *Drosophila melanogaster* cells with and without *Wolbachia* (*w*Mel); Welch-corrected unpaired two-tailed t-test on log-transformed values, SINV (p = 0.0045, df = 9.458, t-ratio = 3.698), CHIKV (t-test, p = 0.013204, df = 10, t-ratio = 3.006), ZIKV (t-test, p = 0.033939, df = 7, t-ratio = 2.629) (S3 Fig). Presence of the same *Wolbachia* genotype in different arthropod hosts thus leads to reduced infectivity of arboviruses belonging to different virus families, indicating that this phenotype is independent of virus type and any specific *Wolbachia-*host association.

We next examined whether different *Wolbachia* strains are capable of reducing progeny virus infectivity in the context of a single arthropod host *(Aedes albopictus)* cell type. A panel of three *Aedes albopictus* derived mosquito cells were obtained, each colonized by a distinct *Wolbachia* strain, including *Wolbachia* strain *w*AlbB (Aa23 cells) derived from the native *Aedes albopictus* and non-native strains *w*Stri (C710 cells) derived from a planthopper, *Laodelphax striatellus* and *w*Mel (RML12 cells), derived from *Drosophila melanogaster* [17]. Phylogenetic analyses of these different *Wolbachia* strains classify them into two distinct clades, with *w*AlbB and *w*Stri representing Supergroup B and *w*Mel Supergroup A (Fig 3C). Historically, the *w*Mel strain has been used successfully in *Wolbachia-*mediated vector control efforts [4, 7]. However, both Supergroup B strains *w*AlbB and *w*Stri are promising candidates that are capable of limiting arbovirus dissemination, given their strong pathogen blocking phenotype in cell culture [10].

All cells, regardless of the *Wolbachia* strain present within, were challenged with SINV at a very low MOI of 0.1 particle/cell to additionally investigate the effect of high (MOI = 10 particle/cell) versus low (MOI = 0.1 particle/cell) starting virus titers on infection outcome in the presence of *Wolbachia*. We found starting MOI to influence progeny SINV infectivity for both W- and W+ viruses, as a lower starting MOI = 0.1 particle/cell (Fig 3D) resulted in overall lower SI ratios at 48 hours post infection relative to higher starting MOI = 10 particles/cell in the same host-*Wolbachia* background (RML12-*w*Mel) (Fig 3B). However, we found SI of progeny W+ SINV to be reduced in each host-*Wolbachia* combination tested, irrespective of the *Wolbachia* strain present (Fig 3D; One-way ANOVA on log-transformed values with Tukey’s post-hoc test; *Wolbachia*, p = 0.00245, *w*Mel, p = 0.0474, *w*AlbB, p = 0.0257, *w*Stri, p = 0.04953). Extent to which SINV infectivity was reduced seemingly varied between cells colonized with different *Wolbachia* strains, which could be a result of either the genotype of the cells, *Wolbachia* strains or both. We observed differences in relative *Wolbachia* titers between the three cell lines. Notably, titers of both Supergroup B strains (*w*AlbB, *w*Stri), that had a more pronounced effect on SINV (Fig 3D), were on average, slightly higher than the Supergroup A *w*Mel strain (RML12 cells) (S4 Fig; Kruskal-Wallis of multivariate comparisons with Dunn’s post-hoc test; RML12-*w*Mel vs C710-*w*Stri, p = 0.3803, RML12-*w*Mel vs Aa23-*w*AlbB, p = 0.0038, Aa23-*w*AlbB vs C710-*w*Stri, p = 0.3803). It is unclear whether these moderate differences in endosymbiont titer are in any way correlated with the extent to which virus infectivity is reduced, given the considerable variation in *Wolbachia* titers across passaged cell lines. Based on these results, we conclude that presence of pathogen-blocking *Wolbachia* strains are commonly associated with a concomitant reduction in progeny virus infectivity.

### Progeny viruses spread poorly in naïve mosquito cells

**Fig 4:**
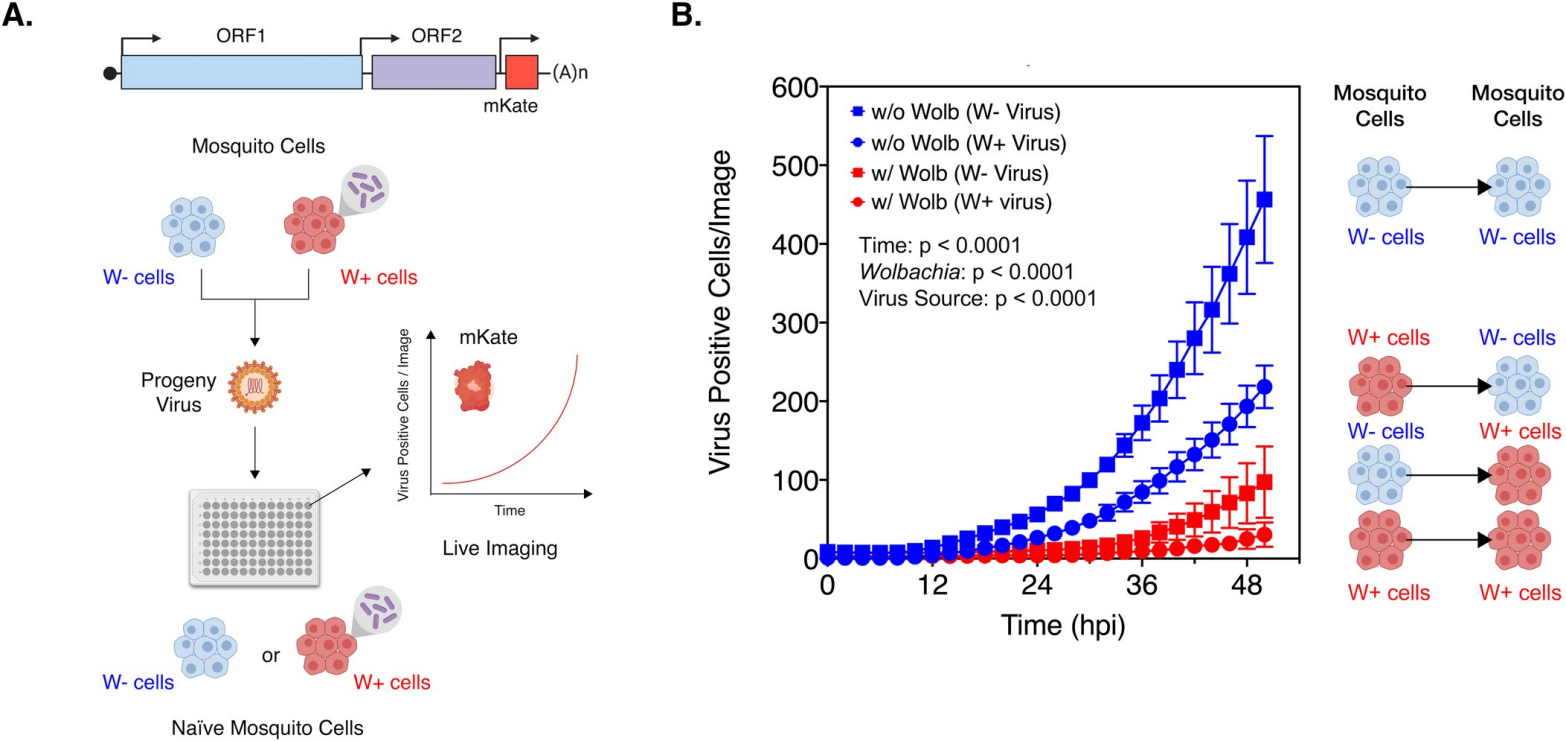
Progeny viruses derived from *Wolbachia* colonized cells replicate poorly in naïve mosquito cells. (A) Schematic representation of the experiment. CHIKV expressing mKate fluorescent protein from a second sub-genomic promoter was grown in C710 *Aedes albopictus* cells in the presence (W+ virus) or absence (W- virus) of *Wolbachia* (*w*Stri strain). These progeny viruses were then used to infect naïve C710 cells with (depicted in red) and without (depicted in blue) *Wolbachia* (*w*Stri strain). synchronously at an MOI of 5 particles/cell. Virus growth in cells, plated on a ninety-six-well plate, was measured in real time by imaging and quantifying the number of red cells (Virus Positive Cells/Image) expressing the virus encoded mKate protein over a period of 48 hours, using live cell imaging. (B) Color of the data points distinguish the two destination cell lines where virus replication was assayed on; blue represent C710 cells without *Wolbachia* while red represent C710 cells with *Wolbachia*. Shape of the data points refer to the nature of the progeny viruses used to initiate infection; squares represent W- viruses, grown in C710 cells without *Wolbachia*, while circles represent W+ viruses, grown in C710 cells with *Wolbachia*. Y-axis label (Virus Positive Cells/Image) represent red cells expressing virus-encoded mKate fluorescent protein in a single field of view, four of which were averaged/sample at every two-hour time point collected over the course of infection. Three-way ANOVA with Tukey’s post-hoc test. Error bars represent standard error of mean (SEM) of biological replicates (n = 9).

We next monitored the ability of progeny viruses derived from mosquito cells colonized with (W+ virus) and without (W- virus) *Wolbachia* to propagate in naïve mosquito cells. CHIKV expressing a fluorescent reporter protein (CHIKV-mKate) was grown in C710 *Aedes albopictus* cells with and without *Wolbachia* (*w*Stri strain), purified and subsequently used to infect naïve mosquito cells at equal MOIs (MOI = 5 particles/cell) following a synchronous infection. Virus infection and spread was then monitored over a period of 50 hours using a live-cell imaging system by quantifying mean virus-encoded fluorescent reporter expression observed over four distinct fields of view taken per well every 2 hours (Fig 4A). Three-way ANOVA was used to determine the effects of virus source (Source i.e. W- or W+ virus), destination cell type (with or without *Wolbachia*) and/or time, on virus spread. Our results show significant effects of all three variables on virus growth, both on their own as well as in combination with each other; Three-way ANOVA with Tukey’s post-hoc test, Source: p < 0.0001, *Wolbachia*: p < 0.0001, Time: p < 0.0001, Source X Time: p < 0.0001, Source X *Wolbachia*: p < 0.0001, *Wolbachia* X Time: p < 0.0001, Source X Time X *Wolbachia*: p = 0.0016 (Fig 4B). As expected, W- virus grew more poorly in naïve cells with *Wolbachia* compared to those without (Fig 4B, square symbols). However, W+ viruses (Fig 4B, circle symbols) exhibited reduced growth in naïve cells both in the presence and absence of *Wolbachia* (*w*Stri strain). Importantly, these results indicate that reduced infectivity of W+ viruses might contribute to their inability to spread to new mosquito cells regardless of whether or not these cells are colonized by *Wolbachia*. Assuming that the mechanism of cellular inhibition of virus replication is cell-autonomous, this model might help explain why limited virus dissemination occurs within tissues with both high and low *Wolbachia* densities. As evident from the results from Fig 4B, our data is consistent with previous observations made by Koh et.al, which reported loss in infectivity of DENV isolates generated following serial passages in *w*Mel-colonized Aag2 cells [6]. Importantly, loss in progeny virus infectivity is not limited to cell culture models. Indeed, as reported by Dutra et.al, in 2016, ZIKV viruses isolated from salivary gland secretions of *w*Mel*-*colonized mosquitoes fail to establish systemic infections in naïve mosquitoes following intrathoracic injection, demonstrating a quantifiable loss in infectivity *in vivo* [7].

### Progeny viruses spread poorly in naïve vertebrate cells

**Fig 5:**
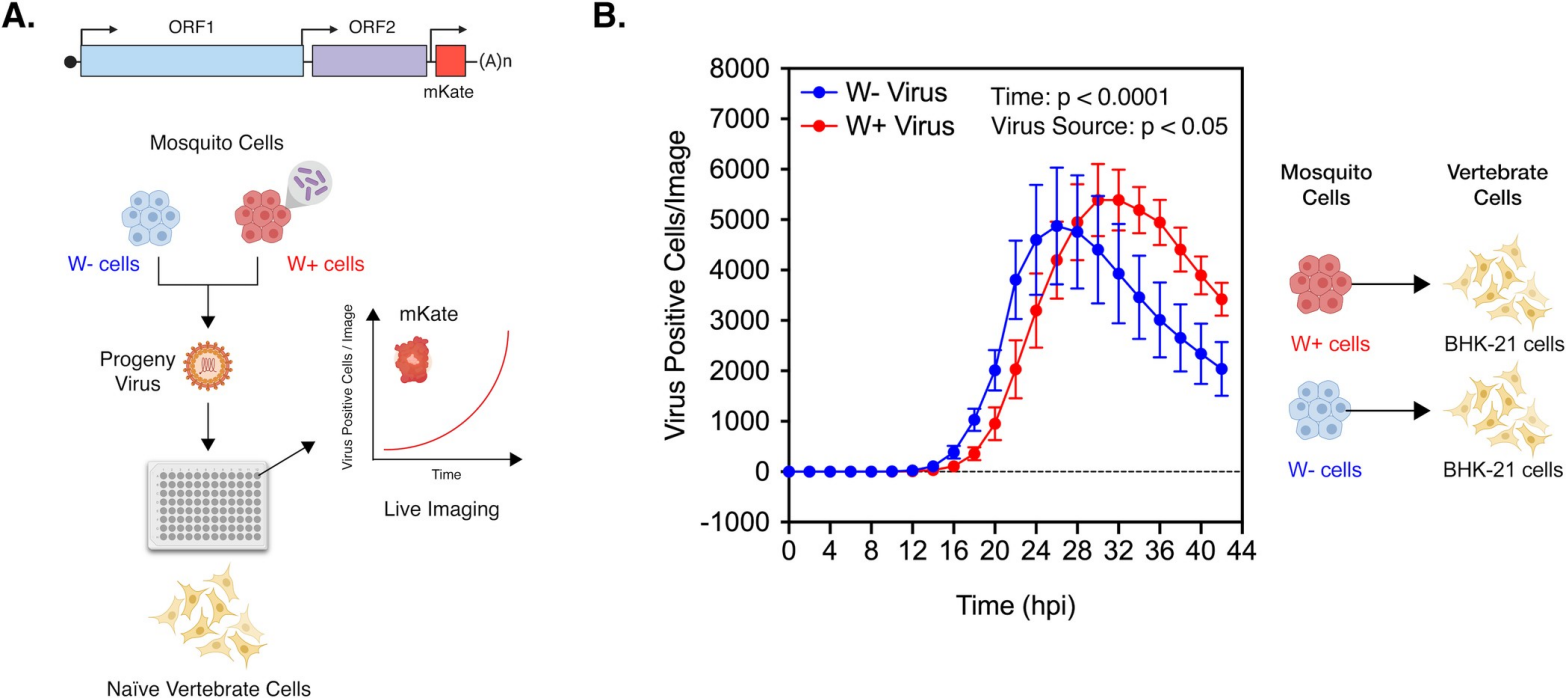
Progeny viruses derived from *Wolbachia* colonized cells replicate poorly in naïve vertebrate cells. **(**A) Schematic representation of the experiment. CHIKV expressing mKate fluorescent protein from a second sub-genomic promoter was grown in C710 *Aedes albopictus* cells in the presence (W+ virus) or absence (W- virus) of *Wolbachia* (*w*Stri strain). These progeny viruses were then used to infect naïve vertebrate BHK-21 cells synchronously at an MOI of 5 particles/cell. Virus growth in cells, plated on a ninety-six-well plate, was measured in real time by imaging and quantifying the number of red cells expressing the virus encoded mKate protein over a period of 42 hours using live-cell imaging. (B) Color of the data points distinguish the progeny viruses used to initiate infection in BHK-cells; blue represent progeny viruses derived from C710 cells without *Wolbachia* (W- virus) while red represent progeny viruses derived from C710 cells with *Wolbachia* (W+ virus). Y-axis label (Virus Positive Cells/Image) represent red cells expressing virus-encoded mKate fluorescent protein in a single field of view, four of which were averaged/sample at every two-hour time point collected over the course of infection. Two-way ANOVA with Tukey’s post-hoc test. Error bars represent standard error of mean (SEM) of biological replicates (n = 3–4).

We further assessed the infectivity of W+ progeny viruses to spread in vertebrate cells using live-cell imaging to validate our earlier results (Fig 3B). As before, purified progeny W+ and W- viruses derived from mosquito cells colonized with (W+ virus) and without (W- virus) *Wolbachia* (*w*Stri strain) was subsequently used to infect naïve BHK-21 cells at an MOI of 5 particles/cell. Infection was synchronized as before and virus spread was measured over 42 hours by quantifying mean virus-encoded fluorescent reporter expression observed over four distinct fields of view taken per well every 2 hours (Fig 5A). Two-way ANOVA was used to determine the effect of virus source (Source i.e. W- or W+ virus) and/or time on virus spread over the course of the infection. Spread of W- viruses were consistently faster compared to W+ viruses, with peak number of W- virus positive cells observed at 26 hours post infection compared to 30 hours post infection for W+ viruses; Ordinary Two-way ANOVA with Tukey’s post-hoc test, Source: p = 0.0261, Time: p < 0.0001, Time X Source: p = 0.0068 (Fig 5B). Interestingly, peak number of virus positive cells was higher for W+ viruses relative to W- viruses between 28 and 42 hours. However, this is due to a delay in cell death in W+ infected cell populations, thus allowing greater and prolonged expression of virus encoded reporter (S5 Fig). In comparison, cells infected with W- viruses succumb early to infection, resulting in a faster loss in reporter activity between 26 and 42 hours (Fig 5B, S5 Fig).

### Encapsidated viral RNA within progeny viruses are less infectious

**Fig 6:**
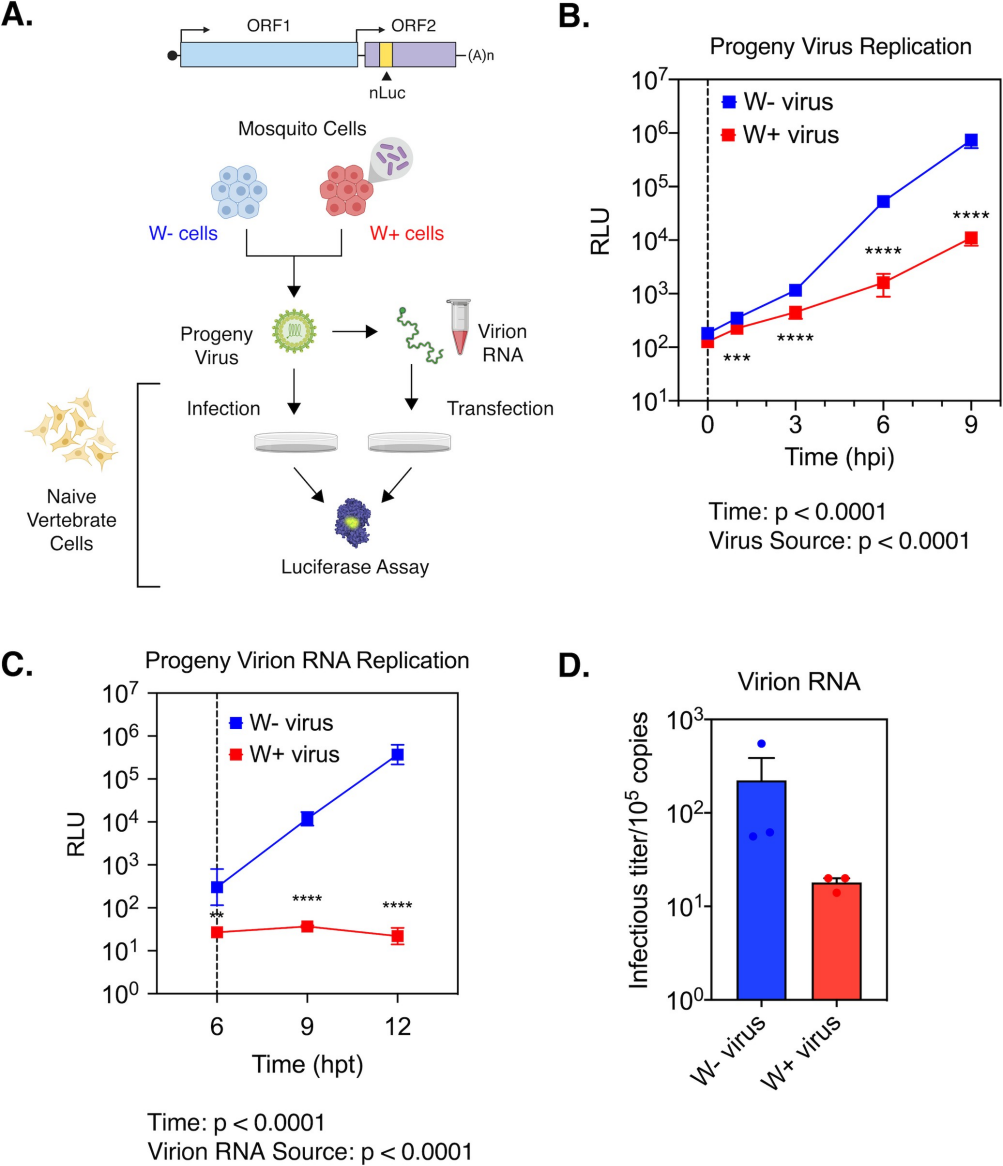
RNA encapsidated within progeny viruses derived from *Wolbachia* colonized cells are less infectious. (A) Schematic of experiments performed using Sindbis Virus (SINV) carrying a translationally fused nanoluciferase (nLuc) gene in the second open reading frame (ORF2). (B) Sindbis nLuc reporter viruses (SINV-nLuc) derived from RML12 mosquito cells with (W+ virus) or without (W- virus) *Wolbachia* (*w*Mel strain) were subsequently used to synchronously infect naïve BHK-21 cells at equivalent MOIs of 5 particles/cell (n = 6). Cell lysates were collected at indicated times post infection and luciferase activity (RLU) was measured and used as a proxy to quantify viral replication. Two-way ANOVA with Tukey’s post-hoc test. Vertical dashed line indicates the first time point collected following removal of the initial inoculum. (C) Replication kinetics of virion encapsidated RNA isolated from progeny SINV-nLuc viruses derived from RML12 mosquito cells with (W+ virus) or without (W- virus) *Wolbachia* (*w*Mel strain) was determined by measuring luciferase activity (RLU) following transfection of 10^5^ copies of virion encapsulated RNA into BHK-21 cells (n = 6). Two-way ANOVA with Tukey’s post-hoc test. Vertical dashed line at 6 hours post transfection (hpt) indicates the first time point collected following removal of the initial transfection mixture. (D) Infectious titer generated from the aforementioned virion RNAs was determined by counting the number of plaques produced after 48 hours post transfection into BHK-21 cells. Error bars represent standard error of mean (SEM) of biological replicates (n = 3). **P < 0.01, ***P < 0.001, ****P < 0.0001.

While reduced infectivity of progeny viruses explains limited virus dissemination and transmission in *Wolbachia*-colonized mosquitoes, it is not evident from our previous data why W+ viruses are less infectious. Simplistically, two key factors can result in the observed loss in virus infectivity; compromised virion structure and dysfunctional viral RNA. The former might impair virus attachment and entry to cells, while the latter would prevent efficient virus replication post entry. We reasoned that if W+ viruses exhibit structural defects that impair their ability to bind and enter cells, direct delivery of the encapsidated viral RNA into the cell should bypass this blockade and thus allow genome replication comparable to W- virus derived RNA. Therefore, we isolated encapsidated virion RNA from W+ and W- viruses produced from *w*Mel-colonized RML12 mosquito cells and assessed their ability to replicate in naïve vertebrate BHK-21 cells following transfection. For this replication assay, we used SINV with nanoluciferase reporter fused to the second open reading frame (SINV-nLuc) and measured luciferase activity as a proxy for viral structural protein expression over time (Fig 6A). First, we used progeny viruses at an MOI of 5 particles/cell to a establish a synchronized infection in BHK-21 cells as described earlier and measured viral replication over a period of 9 hours post infection. We found W+ virus replication to be significantly reduced relative to W- viruses over time, reaffirming our earlier results regarding poor infectivity of W+ viruses on vertebrate cells (Fig 5B); Two-way ANOVA with Tukey’s post-hoc test, Time: p < 0.0001, *Wolbachia*: p < 0.0001, Time X *Wolbachia*, p < 0.0001 (Fig 6B). Next, we isolated virion encapsidated RNA from W+ and W- viruses and transfected naïve BHK-21 cells with equal, approximately 10^5^ viral genome copies (Fig 6A). As before, we used luciferase reporter activity as a proxy for virus replication over time and found significantly reduced reporter activity in cells transfected with W+ virus-derived RNA; Two-way ANOVA with Tukey’s post-hoc test, Time: p < 0.0001, *Wolbachia*: < 0.0001, Time X *Wolbachia*, p < 0.0001 (Fig 6C). This reduction in reporter activity was more severe compared to the reduction observed in our previous experiments where infections were initiated with W+ viruses (Fig 6B). We have previously demonstrated that interactions between Sindbis virus capsid protein and the viral RNA (SINV C: R interaction) are important in regulating the function of the incoming viral RNA in vertebrate cells [16]. Absence of critical SINV C:R interaction sites that impact association of the viral RNA with the capsid proteins lead to increased degradation of the incoming viral RNA, reducing the half-life by almost 2.5 -fold. As SINV C:R interactions are absent during our transfection experiments, it is possible that virion RNA derived from W+ viruses are turned over at a faster rate, causing the observed reduction in replication of W+ virus-derived RNA (Fig 6C). Finally, to test whether reduced replication of W+ virion RNA result in the production of fewer infectious units, we quantified plaque-forming units following transfection of 10^5^ copies of virion isolated RNA into BHK-21 cells and found W+ virion RNA to produce 10-times fewer infectious units relative to W- virion RNA after 48 hours (Fig 6D). We obtained similar results upon transfecting BHK-21 cells with virion RNA isolated from SINV-nLuc W- and W+ viruses derived from JW18 fly cells colonized with *Wolbachia* (*w*Mel strain); Two-tailed Welch’s t-test, p = 0.0196, t = 4.883, df = 2.774 (S6 Fig). Taken together, these results suggest that encapsidated viral RNA present within W+ viruses are deficient in their ability to replicate in naïve vertebrate cells, ultimately resulting in the formation of fewer infectious units.

Considering that viral RNA and protein synthesis is abrogated in arthropod cells in the presence of *Wolbachia*, we conclude based on the data presented above that the viral RNA serves as a shared target for endosymbiont-mediated inhibition in arthropod cells.

## Discussion

Uptake of viruses occur as the mosquito takes an infectious bloodmeal from a vertebrate animal. As the blood meal is digested, these viruses infect midgut cells before escaping the midgut barrier and disseminating to other mosquito tissues, which become persistently infected. Therefore, it is important to note that the viruses initially establishing infection in the vector are of vertebrate origin, while those that undergo dissemination and eventually transmission, are derived from mosquito cells. Prior studies have shown that initial infection of viruses in mosquito midguts is reduced in the presence of *Wolbachia*, suggesting an inability of vertebrate-derived viruses to establish infection [8, 18]. In this study, we show that presence of *Wolbachia* restricts vertebrate cell-derived virus growth in naïve mosquito cells. While past studies have shown inhibition of viral RNA and protein synthesis in *Wolbachia-*colonized cells, it is still unclear how early virus inhibition occurs in cells that are initially infected [13–14]. Using Semilki Forest Viruses (SFV) carrying a translationally fused luciferase reporter, Rainey et.al. demonstrated reduced expression of reporter genes at 7 hours post infection in fly (JW18) cells colonized with *Wolbachia* (*w*Mel strain), suggesting that inhibition occurs early in the infection process [14]. Importantly, the authors also observed reduced luciferase expression following transfection of an *in vitro* transcribed virus replicon reporter, which indicates that inhibition of virus replication is independent of virus entry into *Wolbachia-*colonized cells and therefore might involve an intracellular viral target [13–14]. More recently, Thomas et.al. reported that *Wolbachia-*mediated inhibition of mosquito cell (C6/36) derived DENV-2 in *w*Mel-colonized Aag-2 cells involve degradation of the viral RNA [13]. Interestingly, the authors observe reduced abundance of viral RNA in Aag2-*w*Mel cells as early as 1h post infection, following synchronized binding (4ºC) and internalization (25ºC) of DENV-2 viruses. It is important to note that this reduction likely represent a loss of incoming viral RNA, due to low amounts of nascently synthesized viral RNA being present in the cell this early in infection. Here using metabolic labelling, we determined the half-life of the incoming vertebrate cell-derived viral RNA to show that virus inhibition occurs very early during infection, with the incoming viral RNA undergoing faster turnover in *Wolbachia-*colonized cells. This likely involves one or more cellular RNA degradation pathways, including perhaps orthologs of the RNA exonuclease *Xrn1*, whose role in pathogen blocking of flaviviruses has been demonstrated in *Aedes aegypti* derived Aag2-*w*Mel cells [13]. Taken together, our results agree with past observations of this phenomenon involving other viruses belonging to both *Togaviridae* (SFV) and *Flaviviridae* (DENV-2) families, suggesting perhaps a common mechanism of action across different cell types and RNA viruses [13–14].

Presence of *Wolbachia* in mosquitoes has been shown to also reduce the rate of virus infection in different mosquito tissues. These include tissues proximal to the midgut like Malpighian tubules and fat bodies, as well as distal tissues like salivary glands, implying limited virus dissemination in the presence of the endosymbiont [8]. Interestingly, the degree to which virus inhibition occurs in these tissues is not correlated with either *Wolbachia-*density or its role in innate immunity. In fact, viral RNA levels are dramatically reduced in the mosquito head, which is poorly colonized by *Wolbachia* [8]. These results are confounding, given that the mechanism of pathogen blocking is thought to be cell-autonomous [19]. In addition, virus transmission is demonstrably reduced in *Wolbachia-*colonized mosquitoes [2, 7–9]. It is important to note that in this case, viruses transmitted to a vertebrate animal following a secondary bloodmeal are produced in the mosquito salivary gland tissues. In *Wolbachia-*colonized mosquitoes, reduced transmission is thought to likely occur as a result of reduced virus growth in salivary gland tissues. Interestingly, however, previous studies have found that although salivary gland secretions from *Wolbachia-*colonized mosquitoes contain detectable levels of Zika viral genome copies (ZIKV, Flaviviridae), these levels far exceed the actual number of infectious viruses that can be assayed on either vertebrate or mosquito cells, implying a reduction in per-particle infectivity [7]. Similar results have also been obtained in cell culture for dengue virus (DENV, Flavivirus) [20].

We have previously shown reduced per-particle infectivity of Sindbis virus grown in *Drosophila melanogaster* cells colonized with *w*Mel on vertebrate BHK-21 cells [12]. In this study, we provide evidence that this attribute of pathogen blocking is present in *Aedes albopictus* mosquito cells, colonized with both native (*w*AlbB) and non-native (*w*Mel and *w*Stri) *Wolbachia* strains. Additionally, *Wolbachia* reduces the infectivity of progeny viruses belonging to two distinct families of + ssRNA viruses, *Togaviridae* (SINV, CHIKV) and *Flaviviridae* (ZIKV) in both mosquito and fly cells. Using growth assays we clearly demonstrate the inability of progeny W+ viruses to replicate in naïve vertebrate cells, thus linking virus infectivity to loss in transmission. Importantly, we demonstrate that reduced infectivity of these progeny viruses also limits their ability to grow in naïve mosquito cells. Remarkably, limited virus growth is independent of whether or not these naïve cells are colonized with *Wolbachia*, thus providing an explanation for why viral RNA levels are reduced in tissues with low *Wolbachia* titers. Therefore, while it is unclear whether virus production is abrogated completely from *Wolbachia-*colonized cells, as *Wolbachia* infection rates are known vary significantly between different cell lines and across passages, it is possible that the small proportion of infectious W+ virus progeny is produced from sub-populations of cells with very low/no *Wolbachia*.

While further investigation is required to determine whether these results are limited to single virus type, *Wolbachia* strain and/or host cell species, we expect this phenotype to remain consistent in *Wolbachia-*host associations where pathogen blocking have been reported previously. However, it would also be interesting to explore this phenotype in particular *Wolbachia-*host combinations that lack pathogen blocking abilities against susceptible viruses e.g. Dengue Virus (DENV, Flaviviridae) in *Aedes aegypti* mosquitoes with *w*Pip, West Nile Virus (WNV, Flaviviridae) in *Culex tarsalis* mosquitoes systemically infected with *w*AlbB and Cricket Paralysis Virus (CrPV, Dicistroviridae) in *Drosophila pandora* infected with *w*PanCI [21–23].

Finally, we demonstrate that loss in virus infectivity occur at the level of the encapsidated virion RNA and affects its ability to replicate following direct delivery into vertebrate cells. Further work is required to determine the exact fate of W+ virus derived viral RNA in vertebrate cells in terms of its stability, localization and translation. Given the absence of information regarding potential structural differences between viruses derived from mosquito cells with and without *Wolbachia*, our data does not exclude the possibility of an additional block in progeny virus binding that might exacerbate this inhibitory effect. One notable limitation of our experimental system is the use of a limited number of mammalian epithelial cell lines as a generalized vertebrate model of infection. While cultured fibroblast and epithelial cells like BHK-21 and Vero, respectively, are physiologically relevant and have been regularly used towards the study of alphavirus and flavivirus pathogenesis, further studies using a diverse set of tissue-specific cell lines is required to fully understand the effect of *Wolbachia* on virus transmission [24].

Notably, our findings support the hypothesis of viral RNA being the target for pathogen blocking. *Wolbachia’s* ability to restrict viruses is seemingly limited to positive-sense single-stranded RNA (+ssRNA) viruses, as inhibition of neither negative-sense single-stranded RNA (-ssRNA) nor DNA viruses have been observed either in the field, or under laboratory conditions. It is therefore likely that factor(s) regulating virus inhibition affect this particular genomic feature shared by all susceptible viruses [3, 25–26]. We have previously reported the role of *Drosophila melanogaster* RNA methyltransferase Dnmt2 as a host determinant of pathogen blocking [12]. Interestingly, the mosquito ortholog of Dnmt2 has also been shown to play an important role in pathogen blocking in mosquitoes, albeit in a manner opposite to flies [27]. Furthermore, loss of Dnmt2 levels in fly cells significantly increase viral RNA replication and progeny virus infectivity, implying an antiviral mechanism of action that might involve targeting of the viral RNA. Given the biological role of Dnmt2 as an RNA cytosine methyltransferase, future studies will also focus on exploring the possibility of changes in the methylation profile of viral RNA in the presence and absence of *Wolbachia*.

## Materials and methods

### Insect and Mammalian cell culture

RML12 *Aedes albopictus* cells with and without *Wolbachia w*Mel (a generous gift from Dr. Seth Bordenstein, Vanderbilt University) were grown at 24 ºC in Schneider’s insect media (Sigma-Aldrich) supplemented with 10% (v/v) heat-inactivated fetal bovine serum (Corning), 1% (v/v) each of L-Glutamine (Corning), non-essential amino acids (Corning) and penicillin-streptomycin-antimycotic (Corning). C710 *Aedes albopictus* cells with and without *Wolbachia w*Stri (a generous gift from Dr. Horacio Frydman, Boston University) were grown in 1X Minimal Essential Medium (Corning) supplemented with 5% (v/v) heat-inactivated fetal bovine serum (Corning), 1% (v/v) each of L-Glutamine (Corning), non-essential amino acids (Corning) and penicillin-streptomycin-antimycotic (Corning). Aa23 *Aedes albopictus* cells with and without *Wolbachia w*AlbB (a generous gift from Dr. Horacio Frydman, Boston University) were grown at 24 ºC in Schneider’s insect media (Sigma-Aldrich) supplemented with 20% (v/v) heat-inactivated fetal bovine serum (Corning), 1% (v/v) each of L-Glutamine (Corning), non-essential amino acids (Corning) and penicillin-streptomycin-antimycotic (Corning). JW18 *Drosophila melanogaster* cells with and without *Wolbachia w*Mel were grown at 24 ºC in Shields and Sang M3 insect media (Sigma-Aldrich) supplemented with 10% (v/v) heat-inactivated fetal bovine serum, 1% (v/v) each of L-Glutamine (Corning), non-essential amino acids (Corning) and penicillin-streptomycin-antimycotic (Corning). *Aedes albopictus* C636 cells and mammalian BHK-21 (Baby Hamster Kidney Fibroblast) and Vero (African Green Monkey Kidney Epithelial) cells were grown at either 28ºC (C6/36) or 37 ºC (BHK/Vero) under 5% ambient CO_2_ in 1X Minimal Essential Medium (Corning) supplemented with 10% (v/v) heat-inactivated fetal bovine serum (Corning), 1% (v/v) each of L-Glutamine (Corning), non-essential amino acids (Corning) and penicillin-streptomycin-antimycotic (Corning).

### Virus infection in cells and progeny virus production

Virus stocks were generated from *Aedes albopictus* derived cells (RML12, C710, Aa23) or *Drosophila melanogaster* derived cells (JW18) with or without *Wolbachia* (RML12-*w*Mel, C710-*w*Stri, Aa23-*w*AlbB, JW18-*w*Mel) by infecting naïve cells with virus at an MOI of 10 particles/cell. In all cases, serum-free media was used for downstream virus purification. Media containing virus was collected 5 days post-infection for alphaviruses SINV (SINV-nLuc), CHIKV (CHIKV18125-capsid-mKate), and 7 days post-infection for flavivirus ZIKV (MR766 Uganda Strain). Virus stocks were subsequently purified and concentrated by ultracentrifugation (43K for 2.5 h) over a 27% (w/v) sucrose cushion dissolved in HNE buffer (20mM HEPES, 0.15M NaCl, 0.1mM EDTA). Viral pellets were stored and aliquoted in HNE buffer before being used for all subsequent experiments. Viral titers were determined using standard plaque assay on vertebrate cells (BHK-21 cells for SINV, CHIKV and Vero cells for ZIKV). Cells were fixed either 48 hours post infection (SINV, CHIKV) or 72 hours post infection (ZIKV) using 10% (v/v) formaldehyde and stained with crystal violet to visualize plaques. Virus particles were determined by quantifying viral genome copies via quantitative RT-PCR using primers listed in the primer table (S1 Table) and standard curves comprised of linearized infectious clone sequences containing either full-length viral genomes (SINV, CHIKV) or partial Env gene (ZIKV).

### Mosquito rearing and infectious blood meals

*Aedes aegypti* mosquitoes either -infected and -uninfected with *Wolbachia* (*w*AlbB strain) (generously provided by Dr. Zhiyong Xi, Michigan State University, USA), were reared in an insect incubator (Percival Model I-36VL, Perry, IA, USA) at 28°C and 75% humidity with 12 h light/dark cycle. Four to six-day old mated female mosquitoes were allowed to feed for 1h on approximately 10^8^ PFUs of SINV (TE12-untagged) containing citrated rabbit blood (Fisher Scientific DRB030) supplemented with 1mM ATP (VWR) and 10% sucrose using a Hemotek artificial blood feeding system (Hemotek, UK) maintained under constant temperature of 37°C. Engorged mosquitoes were then isolated and reared at 28°C in the presence of male mosquitoes. Infected mosquitoes were harvested 5–7 days post blood meal before being snap frozen in liquid nitrogen and stored at −80°C before further processing. Samples for qPCR and qRT-PCR were homogenized in TRiZOL (Sigma Aldrich) reagent and further processed for nucleic acid extractions using manufacturer’s protocols.

### Virion RNA extraction and transfection

Virion encapsidated RNA was extracted from viruses (SINV-nLuc) were purified over a 27% sucrose cushion using TRiZOL reagent (Sigma Aldrich) using manufacturer’s protocol. Post extraction, RNAs were DNase (RQ1 RNase-free DNase, NEB) treated using manufacturer’s protocol to remove cellular contaminants and viral RNA copies were quantified via quantitative RT-PCR using primers probing for SINV nsP1 and E1 genomic regions (Supplementary S1 Table) and a standard curve comprised of linearized SINV infectious clone containing the full-length viral genome. To determine infectivity or replication kinetics of Sindbis virion RNA, equal copies of virion isolated RNA (10^5^ copies), quantified using qRT-PCR, were transfected into BHK-21 cells in serum-free Opti-MEM (Gibco). To maximize production of infectious units, equal mass (1 μg) of virion (SINV-nLuc) isolated RNA derived from JW18 fly cells was transfected into BHK-21 cells. Transfection was carried out for 6 hours before the transfection inoculum was removed and overlay was applied. Cells were fixed 48 hours post transfection using 10% (v/v) formaldehyde and stained with crystal violet to visualize plaques.

### Live cell imaging

Growth of fluorescent reporter viruses in mosquito (C710) and vertebrate (BHK-21) cells were monitored using Incucyte live cell analysis system (Essen Biosciences, USA). Cells were grown under standard conditions as described earlier under 5% ambient CO_2_ either at 37ºC for vertebrate BHK-21 cells and 27ºC for *Aedes albopictus* C710 cells. Cells were plated to 75–80% confluency in 96-well plates to allow distinct separation between adjacent cells and preserve cell shape for optimal automated cell counting. Cells per well were imaged and averaged across four distinct fields of view, each placed in one quarter of the well, every 2 hours over the course of the infection. For every sample, total fluorescence generated by cells expressing the red fluorescent reporter mKate was calculated and normalized by the cell number. A manual threshold was set to minimize background signal via automated background correction at the time of data collection. Following acquisition, data was analyzed real-time using the native Incucyte Base Analysis Software.

### Incoming viral genomic RNA half-life assay

C710 *Aedes albopictus* cells with (w/ *w*Stri) or without *Wolbachia* (w/o *w*Stri) and JW18 *Drosophila melanogaster* cells with (w/ *w*Mel) or without *Wolbachia* (w/o *w*Mel) were grown overnight (~12h) in media supplemented with 50 μM nucleoside analog 4-thiouridine (Sigma). These 4SU-treated cells were synchronously infected with Sindbis virus (SINV) derived from vertebrate BHK-21 cells at an MOI of 10 particles/cell. At indicated times post infection, tissue culture supernatants were removed, and cell monolayers were washed with 1XPBS prior to RNA extraction using TRiZOL reagent (Sigma Aldrich) using manufacturer’s protocols. A total of 1μg of total RNA per sample was biotinylated using HPDP-Biotin (Pierce) and all 4SU-labelled RNA was separated from the total RNA pool using streptavidin cleanup and levels of unlabeled RNA at each time point was measured relative to that present at 0 minutes post internalization. RNA half-life was extrapolated using non-linear, exponential decay model as described previously [16, 28].

### Viral replication assays

Quantification of viral protein translation was performed using cellular lysates following synchronized infections with reporter viruses (SINV-nLuc), or transfections with virion-derived RNA from the aforementioned viruses. At indicated times post infection, samples were collected and homogenized in 1X Cell Culture Lysis Reagent (Promega). Samples were mixed with NanoGlo luciferase reagent (Promega), incubated at room temperature for 3 minutes before luminescence was recorded using a Synergy H1 microplate reader (BioTech instruments).

### Real-time quantitative PCR and RT-PCR analyses

Total DNA and RNA from samples were extracted using TRiZOL reagent (Sigma Aldrich) using manufacturer’s protocols. Complementary DNA (cDNA) was synthesized using MMuLV Reverse Transcriptase (NEB) with random hexamer primers (Integrated DNA Technologies). Negative (no RT or no gDNA) controls were performed for each target. Quantitative PCR or RT-PCR analyses were performed using Brilliant III SYBR green QPCR master mix (Bioline) with gene-specific primers according to the manufacturer's protocol and with the Applied Bioscience StepOnePlus qPCR machine (Life Technologies). All primer sets, except for ZIKV_MR766 Env and CHIKV_18125 E2 were designed based on information present in existing literature [15, 20]. For primers designed specifically for this study, primer amplification efficiency was calculated using standard curves (see S1 Table for more details). The expression levels were normalized to the endogenous 18S rRNA expression using the delta-delta comparative threshold method (ΔΔCT) (S1 Table).

### Phylogenetic analyses

MUSCLE alignment was performed on concatenated *Wolbachia* sequences comprising five MLST genes *coxA*, *gatB*, *ftsZ*, *hcpA* and *fbpA*. The resulting alignment was used to perform Maximum-likelihood analyses in MEGA X with a bootstrap value set to 1000. The resulting newick (.nwk) file was used to construct a phylogenetic tree using FigTree v1.4.4.

### Statistical analyses of experimental data

All statistical analyses were conducted using GraphPad Prism 8 (GraphPad Software Inc., San Diego, CA).

## Supporting information

S1 Fig Viral RNA levels are reduced *in vivo* and in mosquito cell culture. (A) Viral RNA levels were quantified in individual adult female *Aedes aegypti* mosquitoes with and without *Wolbachia* (*w*AlbB strain) using qRT-PCR at 7-days post infectious blood meal with SINV. Error bars represent standard error of mean (SEM) of biological replicates (n = 6). Welch’s t-test performed on log-transformed values. *P < 0.05 (B) Viral RNA replication in mosquito cells colonized with *Wolbachia*. RML12 mosquito cells with or without *Wolbachia* (*w*Mel strain) were infected with SINV at an MOI of 10 particles/cell. Total cellular RNA was harvested 48 hours post infection and assayed for viral RNA levels using quantitative RT-PCR. Error bars represent standard error of mean (SEM) of biological replicates (n = 6). Welch’s t-test performed on log-transformed values. ****P < 0.0001.
(TIF)

S2 Fig Mean percentage of total CHIKV (A) and ZIKV (B) particles produced from *Aedes albopictus* RML12 cells colonized with (w/ *Wolb*) or without (w/o *Wolb*) *Wolbachia* (*w*Mel strain) (n = 6). Corresponding specific infectivity ratios are presented in Fig 3B.
(TIF)

S3 Fig Specific Infectivity Ratios of progeny RNA viruses Sindbis (SINV), Chikungunya (CHIKV) and Zika (ZIKV) derived from *Drosophila melanogaster* JW18 cells colonized with the native *Wolbachia* strain *w*Mel. Welch’s t-test performed on log-transformed values. Error bars represent standard error of mean (SEM) of biological replicates (n = 5–6). *P < 0.05; **P < 0.01.
(TIF)

S4 Fig Relative *Wolbachia* titer in arthropod cell-lines used in the study was quantified using quantitative PCR. Kruskal-Wallis test of multivariate comparisons with Dunn’s post-hoc test. Error bars represent standard error of mean (SEM) of biological replicates (n = 4). **P < 0.01, ns = non-significant.
(TIF)

S5 Fig Live cell imaging was performed using the Incucyte platform (Essen Biosciences) over a time course of 44 hours in vertebrate BHK-21 cells following synchronized infection of mKate-expressing fluorescent virus (CHIKV-mKate) derived from C710 *Aedes albopictus* cells colonized with or without *w*Stri strain of *Wolbachia* at an MOI of 5 particles/cell. Micrograph images presented here represent of one out of four fields of view collected per replicate/time point (n = 6) every 4 hours (A-L). For each set, left and right images represent BHK-21 cells infected with W- virus (derived from C710 cells without wStri) and W+ virus (derived from C710 cells with wStri) respectively. Infected cell populations are visible as red cells, outlines of which are masked in blue to allow automated quantification using Incucyte Base Analysis Software (quantified in Fig 5).
(TIF)

S6 Fig Infectious titer generated in vertebrate BHK-21 cells following transfection of virion encapsidated RNA isolated from progeny SINV-nLuc W- and W+ viruses. Viruses were derived from JW18 fly cells with (W+ virus) or without (W- virus) *Wolbachia* (*w*Mel strain). 1 μg of total virion RNA was transfected into BHK-21 cells and infectious titer was determined by counting the number of plaques produced after 48 hours post transfection. Details of the assay can be found in Materials and Methods. Error bars represent standard error of mean (SEM) of biological replicates (n = 3). Welch’s t-test on log-transformed data. *P < 0.05.
(TIF)

S1 Table Primers used in the study were purchased from Integrated DNA Technologies (IDT). All primers were used at a final concentration of 10μM for quantitative RT-PCR reactions. All primer sets, except for ZIKV_MR766 Env and CHIKV_18125 E2 were designed based on information present in existing literature [15, 20]. For primer sets designed specifically for this study, primer amplification efficiency was carried out using standard curves. (CHIKV_18125 E2: Slope = -0.69, Amplification factor = 4.90, Efficiency ~ 100%, ZIKV_MR766 Env: Slope = -0.761, Amplification factor = 5.77, Efficiency ~ 100%).
(DOCX)
