# Supplementary figures and images for: Viral RNA is a target for Wolbachia-mediated pathogen blocking
Source: PLoS Pathog. 2020 Jun 18;16(6):e1008513. doi: 10.1371/journal.ppat.1008513 (PMC7326284; doi:10.1371/journal.ppat.1008513)

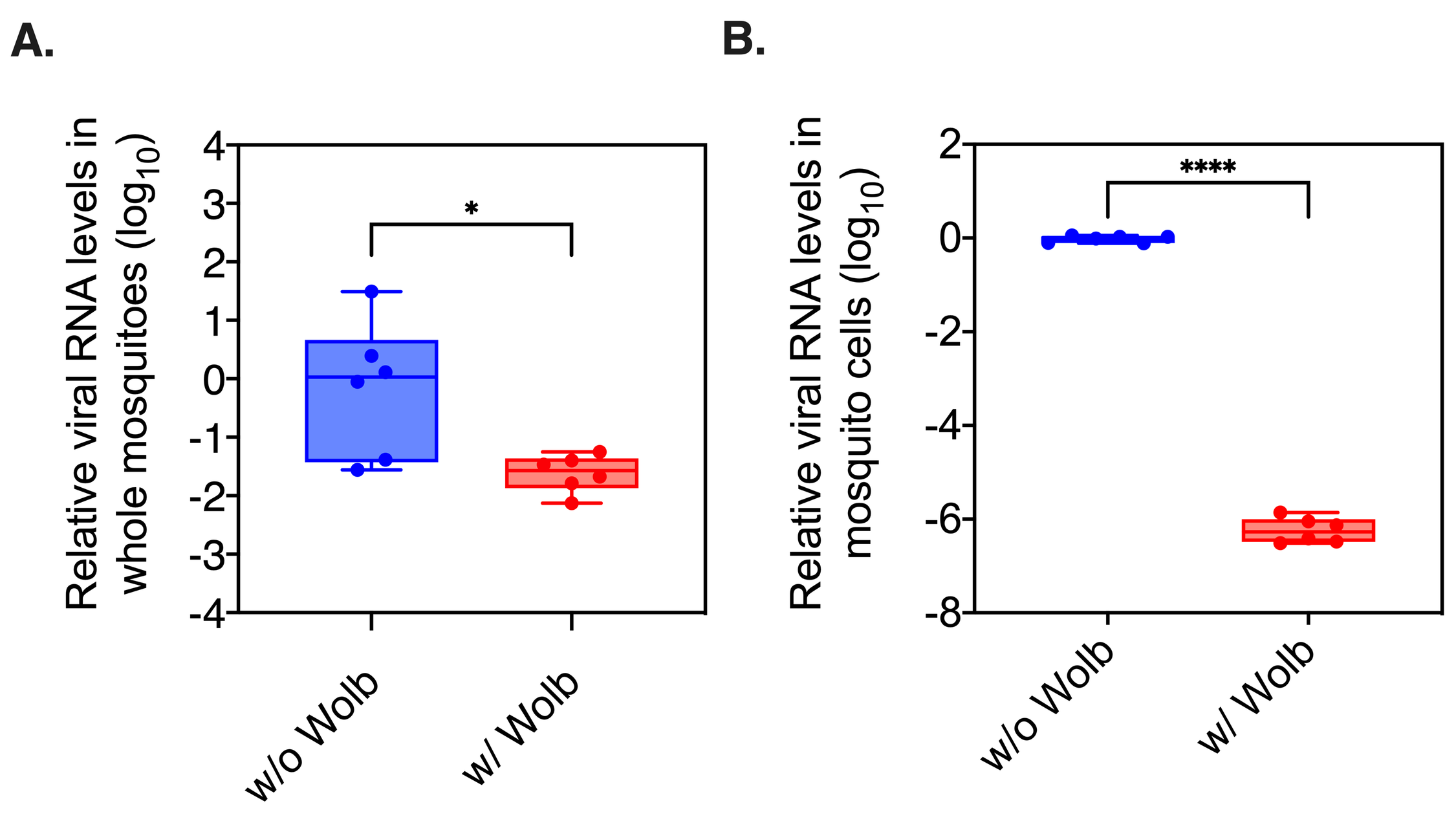

Supplement: S1 Fig — (A) Viral RNA levels were quantified in individual adult female Aedes aegypti mosquitoes with and without Wolbachia (wAlbB strain) using qRT-PCR at 7-days post infectious blood meal with SINV. Error bars represent standard error of mean (SEM) of biological replicates (n = 6). Welch’s t-test performed on log-transformed values. *P < 0.05 (B) Viral RNA replication in mosquito cells colonized with Wolbachia. RML12 mosquito cells with or without Wolbachia (wMel strain) were infected with SINV at an MOI of 10 particles/cell. Total cellular RNA was harvested 48 hours post infection and assayed for viral RNA levels using quantitative RT-PCR. Error bars represent standard error of mean (SEM) of biological replicates (n = 6). Welch’s t-test performed on log-transformed values. ****P < 0.0001. (TIF) [file ppat.1008513.s001.tif]

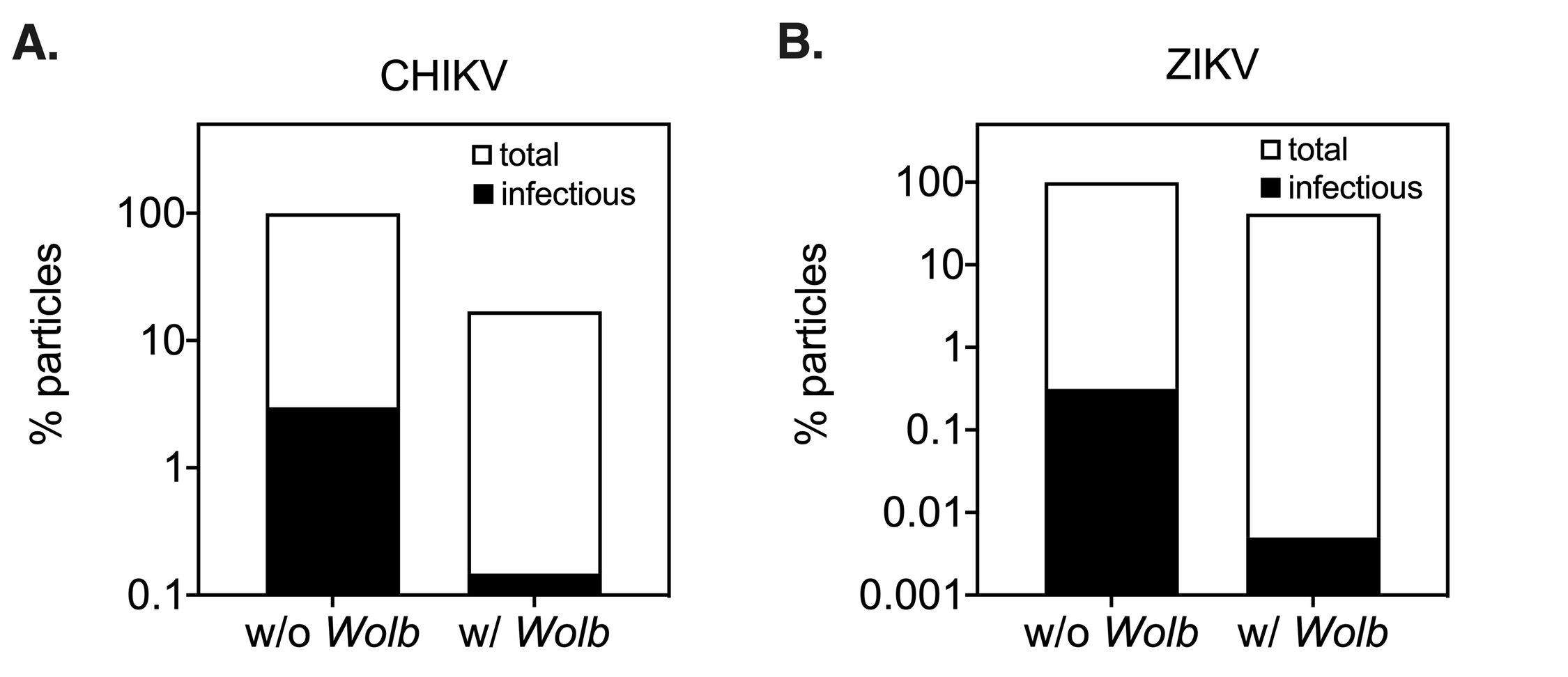

Supplement: S2 Fig — Mean percentage of total CHIKV (A) and ZIKV (B) particles produced from Aedes albopictus RML12 cells colonized with (w/ Wolb) or without (w/o Wolb) Wolbachia (wMel strain) (n = 6). Corresponding specific infectivity ratios are presented in Fig 3B. (TIF) [file ppat.1008513.s002.tif]

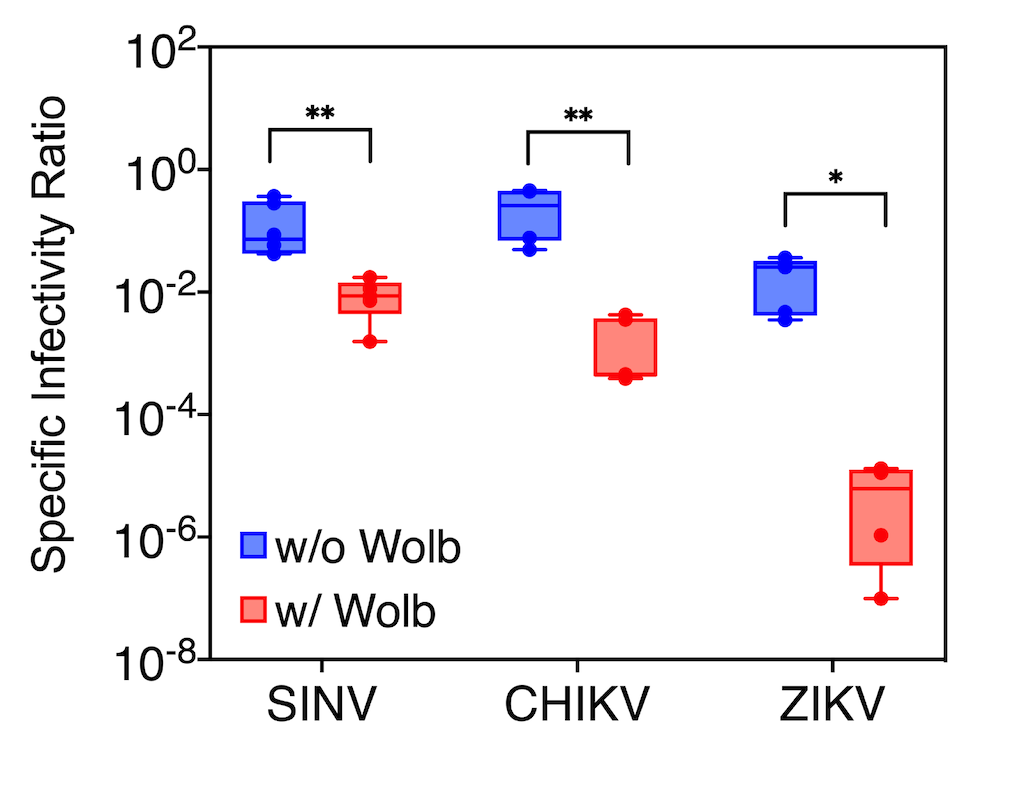

Supplement: S3 Fig — Welch’s t-test performed on log-transformed values. Error bars represent standard error of mean (SEM) of biological replicates (n = 5–6). *P < 0.05; **P < 0.01. (TIF) [file ppat.1008513.s003.tif]

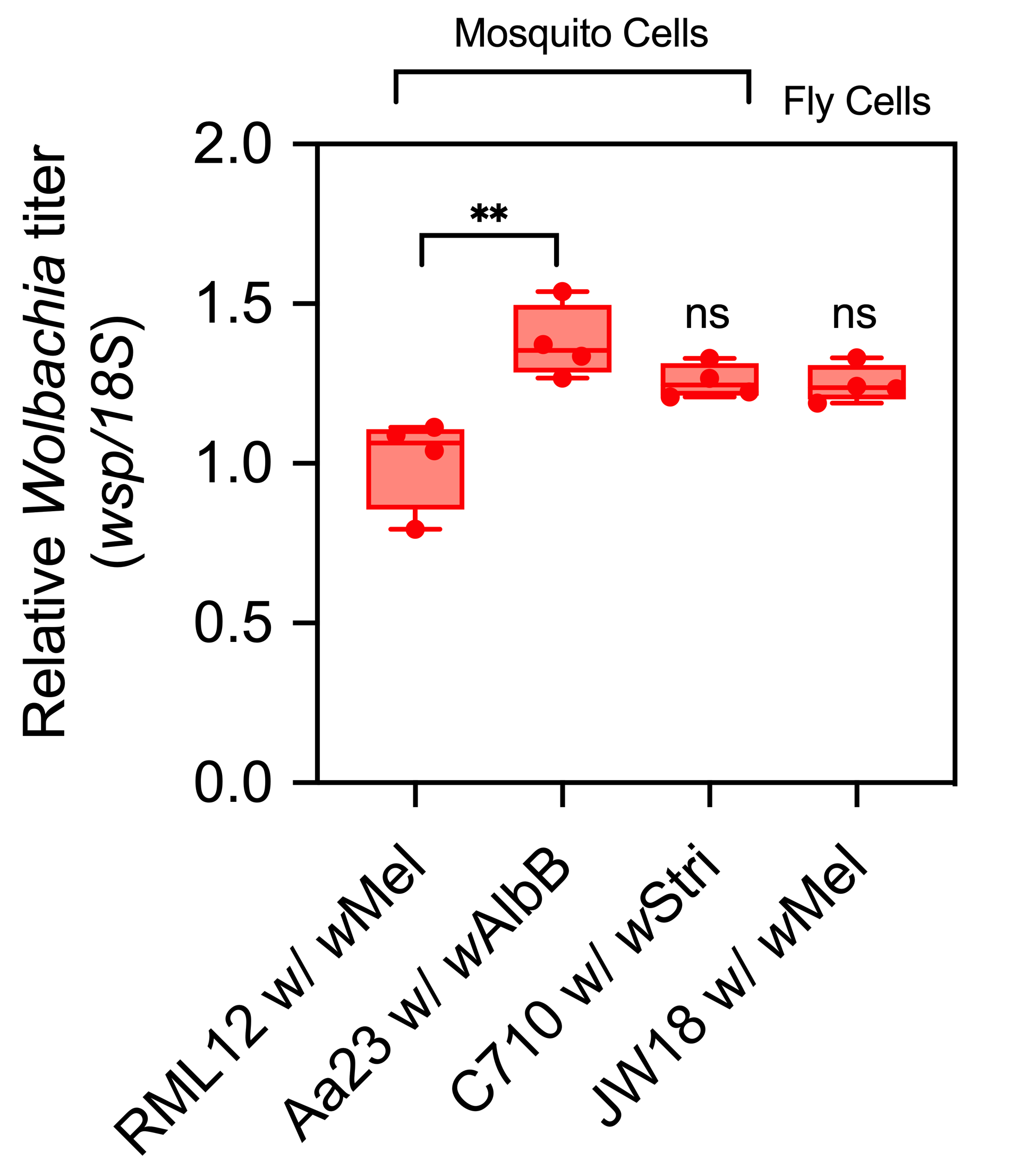

Supplement: S4 Fig — Kruskal-Wallis test of multivariate comparisons with Dunn’s post-hoc test. Error bars represent standard error of mean (SEM) of biological replicates (n = 4). **P < 0.01, ns = non-significant. (TIF) [file ppat.1008513.s004.tif]

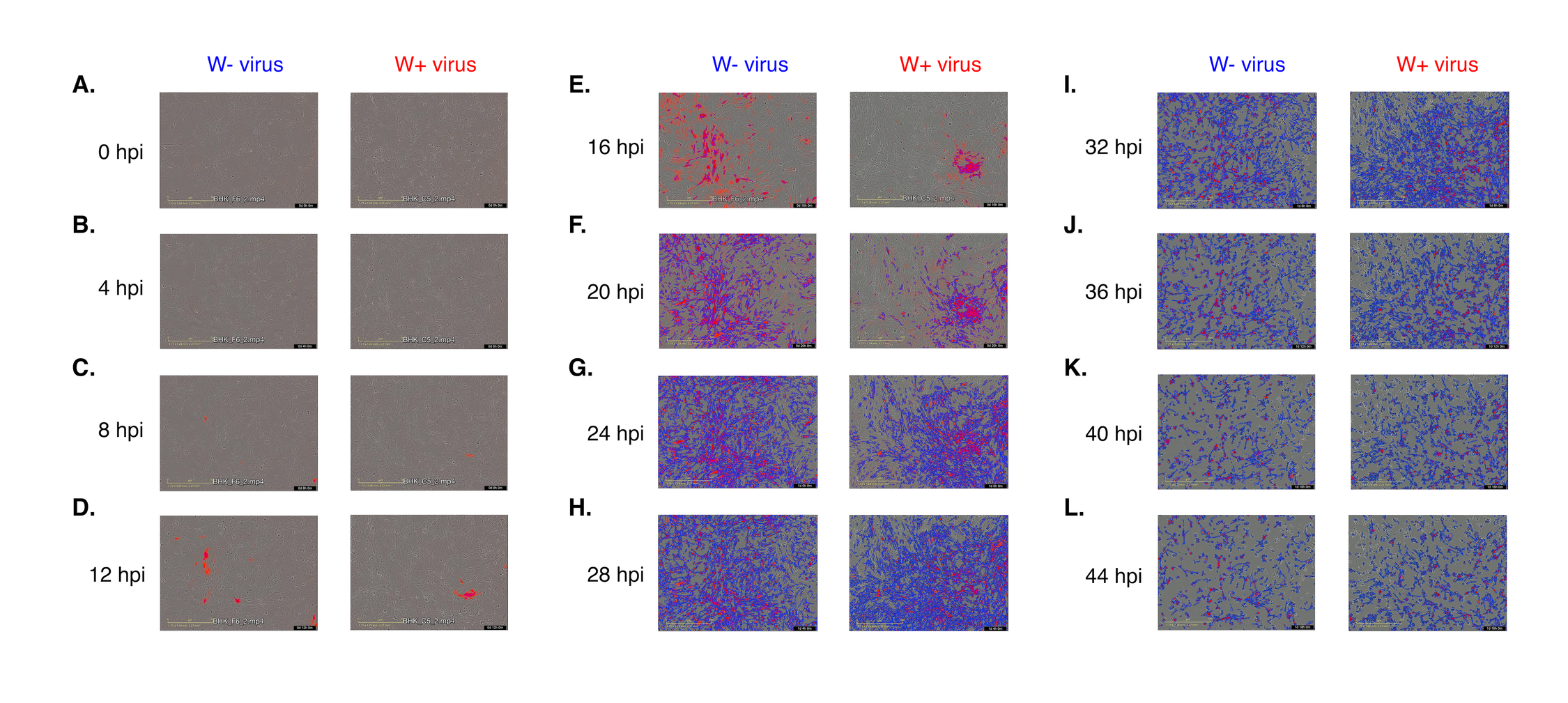

Supplement: S5 Fig — Micrograph images presented here represent of one out of four fields of view collected per replicate/time point (n = 6) every 4 hours (A-L). For each set, left and right images represent BHK-21 cells infected with W- virus (derived from C710 cells without wStri) and W+ virus (derived from C710 cells with wStri) respectively. Infected cell populations are visible as red cells, outlines of which are masked in blue to allow automated quantification using Incucyte Base Analysis Software (quantified in Fig 5). (TIF) [file ppat.1008513.s005.tif]

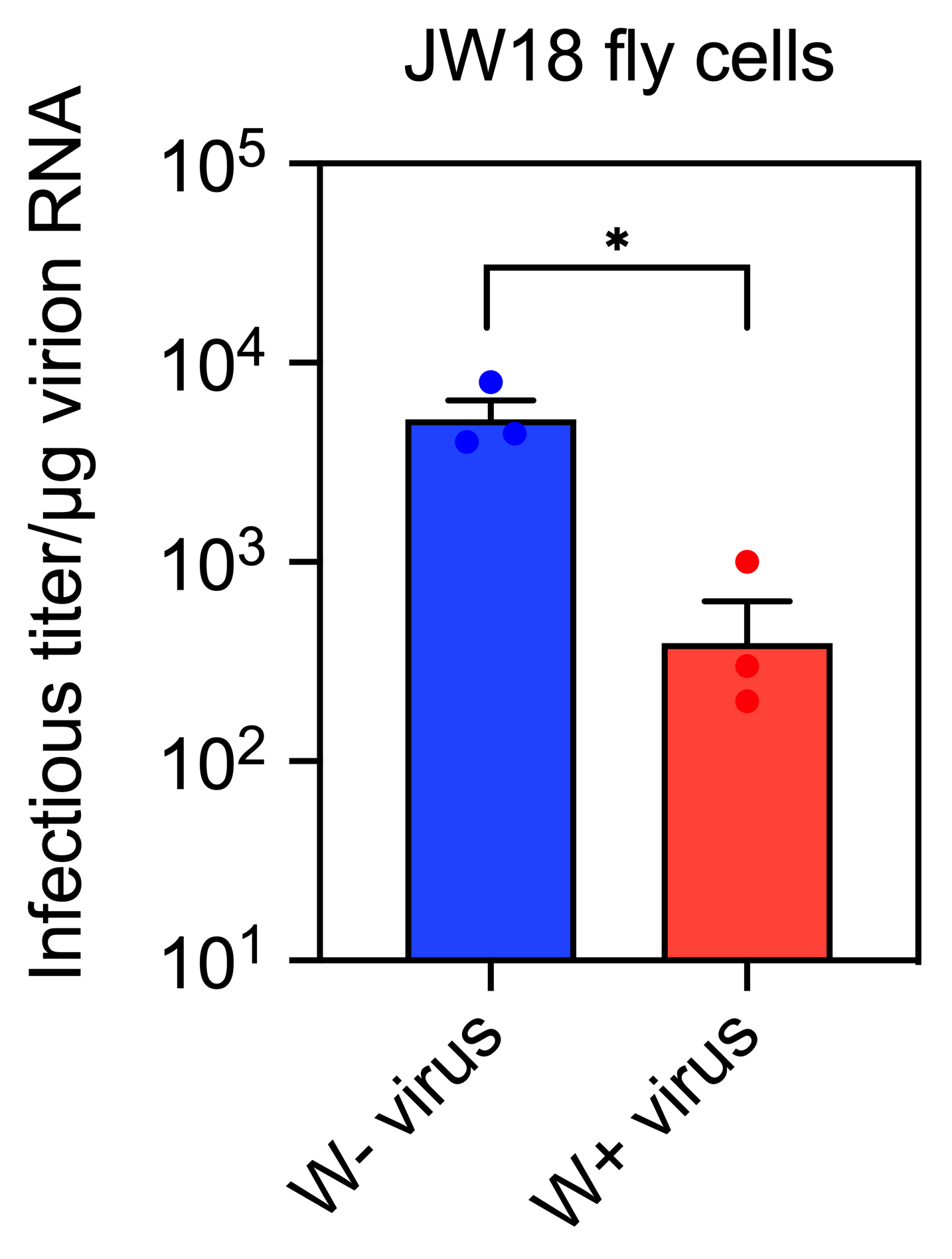

Supplement: S6 Fig — Viruses were derived from JW18 fly cells with (W+ virus) or without (W- virus) Wolbachia (wMel strain). 1 μg of total virion RNA was transfected into BHK-21 cells and infectious titer was determined by counting the number of plaques produced after 48 hours post transfection. Details of the assay can be found in Materials and Methods. Error bars represent standard error of mean (SEM) of biological replicates (n = 3). Welch’s t-test on log-transformed data. *P < 0.05. (TIF) [file ppat.1008513.s006.tif]
