# Supplementary material for: Viral RNA is a target for Wolbachia-mediated pathogen blocking
Source: PLoS Pathog. 2020 Jun 18;16(6):e1008513. doi: 10.1371/journal.ppat.1008513 (PMC7326284; doi:10.1371/journal.ppat.1008513)
Supplement: S1 Table — All primers were used at a final concentration of 10μM for quantitative RT-PCR reactions. All primer sets, except for ZIKV_MR766 Env and CHIKV_18125 E2 were designed based on information present in existing literature [15, 20]. For primer sets designed specifically for this study, primer amplification efficiency was carried out using standard curves. (CHIKV_18125 E2: Slope = -0.69, Amplification factor = 4.90, Efficiency ~ 100%, ZIKV_MR766 Env: Slope = -0.761, Amplification factor = 5.77, Efficiency ~ 100%). (DOCX) [file ppat.1008513.s007.docx]

| **Primer Name** | **Forward Primer Sequence (5’-3’)** | **Reverse Primer Sequence (5’-3’)** |
| --- | --- | --- |
| SINV nsP1 | AAGGATCTCCGGACCGTACTTG | CATGAACTGGGTGGTGTCGAAGC |
| SINV E1 | TCAGATGCACCACTGGTCTCAACA | ATTGACCTTCGCGGTCGGATACAT |
| CHIKV_18125 E2 | GGAATAAAGACGGATGATAGC | GGTCGGGAATGAAATTTTTCC |
| ZIKV_MR766 Env | GGAACTCCACACTGGAACAA | ACCATCCATCTCAGCCTCTA |
| 18S | CGAAAGTTAGAGGTTCGAAGGCGA | CCGTGTTGAGTCAAATTAAGCCGC |
| WSP | CATTGGTGTTGGTGTTGGTG | ACCGAAATAACGAGCTCCAG |
